# Baseline PI susceptibility by HIV-1 Gag-protease phenotyping and subsequent virological suppression with PI-based second-line ART in Nigeria

**DOI:** 10.1093/jac/dkz005

**Published:** 2019-02-04

**Authors:** R Datir, K El Bouzidi, P Dakum, N Ndembi, R K Gupta

**Affiliations:** 1 Division of Infection and Immunity, University College London, London, UK; 2 Institute of Human Virology, Abuja, Nigeria; 3 Africa Health Research Institute, Durban, South Africa

## Abstract

**Objectives:**

Previous work showed that *gag-protease*-derived phenotypic susceptibility to PIs differed between HIV-1 subtype CRF02_AG/subtype G-infected patients who went on to successfully suppress viral replication versus those who experienced virological failure of lopinavir/ritonavir monotherapy as first-line treatment in a clinical trial. We analysed the relationship between PI susceptibility and outcome of second-line ART in Nigeria, where subtypes CRF02_AG/G dominate the epidemic.

**Methods:**

Individuals who experienced second-line failure with ritonavir-boosted PI-based ART were matched (by subtype, sex, age, viral load, duration of treatment and baseline CD4 count) to those who achieved virological response (‘successes’). Successes were defined by viral load <400 copies of HIV-1 RNA/mL by week 48. Full-length Gag-protease was amplified from patient samples for *in vitro* phenotypic susceptibility testing, with PI susceptibility expressed as IC_50_ fold change (FC) relative to a subtype B reference strain.

**Results:**

The median (IQR) lopinavir IC_50_ FC was 4.04 (2.49–7.89) for virological failures and 4.13 (3.14–8.17) for virological successes (*P *=* *0.94). One patient had an FC >10 for lopinavir at baseline and experienced subsequent virological failure with ritonavir-boosted lopinavir as the PI. There was no statistically significant difference in single-round replication efficiency between the two groups (*P *=* *0.93). There was a moderate correlation between single-round replication efficiency and FC for lopinavir (correlation coefficient 0.32).

**Conclusions:**

We found no impact of baseline HIV-1 Gag-protease-derived phenotypic susceptibility on outcomes of PI-based second-line ART in Nigeria.

## Introduction

Prevalence of virological failure for first-line antiretroviral therapy can be as high as 30%,^1^ with high-level resistance to NNRTI, tenofovir and cytosine analogues common in resource-limited settings and compounded by prior undisclosed ART.^2^^,^^3^ Second-line ART recommended by WHO comprises a ritonavir-boosted PI and two NRTIs, commonly lopinavir or atazanavir.^4^ PIs are the second- and last-line therapy for the majority of HIV-infected patients worldwide as access to third-line therapy is still limited.^5^ Virological failure with PIs as second-line therapy occurs in around 20% of individuals.^6–8^ In contrast to first-line therapy, with which >80% develop drug resistance mutations, only around 10%–20% develop major resistance mutations to PIs by week 48,^6^^,^^7^^,^^9^^,^^10^ and this proportion increases over time.^5^

It is known that proteins such as Gag and Env can affect susceptibility to PIs even in the absence of known major resistance mutations in the *protease* gene.^11–15^ There are limited data on changes in *gag* following treatment failure with PIs in the non-B subtypes that dominate low- and middle-income countries.^15–20^ It appears that in around 15% of patients failing boosted PI (bPI) without major protease mutations, a decrease in phenotypic susceptibility to the drug appears to occur when *gag-protease* is phenotyped.^21–23^ Therefore it is conceivable that underlying phenotypic susceptibility resulting from variation in genes such as *gag* and *env* might impact clinical responses to PI.

We previously showed that *gag-protease*-derived phenotypic susceptibility differed between CRF02_AG and subtype G-infected patients who went on to successfully suppress viral replication versus those who experienced virological failure (VF) of lopinavir/ritonavir monotherapy as first-line treatment in a clinical trial.^12^ In order to determine the relevance of this finding for real-world settings in the context of tenofovir disoproxil fumarate + lamivudine or zidovudine + lamivudine with ritonavir-boosted PI (lopinavir or atazanavir) we analysed the relationship between PI susceptibility and the outcome of PI-based second-line ART in Nigeria, where subtypes CRF02_AG and G dominate the epidemic.^24^

## Patients and methods

### Study participants

This study involved retrospectively testing samples from patients attending for HIV care at University of Abuja Teaching Hospital (UATH) who experienced second-line failure (HIV-1 RNA >1000 copies/mL after >6 months on treatment) on a lopinavir/ritonavir- or atazanavir/ritonavir-containing regimen, without any major PI mutations, who were selected as ‘cases’. They were matched to ‘controls’, who had achieved virological suppression lasting up to 12 months (HIV-1 RNA <400 copies/mL) with a similar age, sex, baseline CD4 count and duration of treatment. Baseline (pre-PI) plasma samples from these matched pairs were retrospectively retrieved.

### Amplification of full-length gag-protease genes

HIV-1 RNA was manually extracted from archived plasma samples using the QIAamp viral RNA extraction kit. Using previously described techniques,^11^^,^^25^ full-length *gag-protease* was amplified and cloned into a subtype B-based (p8.9NSX+) vector. Clonal sequencing of up to 10 plasmids (where possible) was performed by standard Sanger sequencing. The variant that most closely represented the consensus (obtained via next-generation sequencing as previously described^6^) was taken forward for phenotypic testing. Sequences were manually analysed using DNA dynamo software (http://www.bluetractorsoftware.co.uk) and MEGA v7.0 software.^26^ Protease sequences were analysed for PI resistance mutations using the Stanford Resistance Database (https://hivdb.stanford.edu).

### PI susceptibility and infectivity assays

PI susceptibility and viral infectivity were determined using a previously described single assay. Briefly, 293T cells were co-transfected with a Gag-Pol protein expression vector (p8.9NSX+) containing cloned patient-derived *gag-protease* sequences, pMDG expressing vesicular stomatitis virus envelope glycoprotein (VSV-g), and pCSFLW (expressing the firefly luciferase reporter gene with HIV-1 packaging signal).

PI drug susceptibility testing was carried out as previously described.^25^ Transfected cells were seeded with serial dilutions of lopinavir and harvested pseudovirions were used to infect fresh 293T cells. To determine strain infectivity, transfected cells were seeded in the absence of drug. Infectivity was monitored by measuring luciferase activity 48 h after infection. Results derived from at least two independent experiments (each in duplicate) were analysed. The IC_50_ was calculated using GraphPad Prism 5 (GraphPad Software Inc., La Jolla, CA, USA). Susceptibility was expressed as a fold change in IC_50_ compared with the subtype B reference strain (p8.9NSX+). Replicative capacity of these viruses was assessed by comparing the luciferase activity of recombinant virus with that of the WT subtype B control virus in the absence of drug. Equal amounts of input plasmid DNA were used, and it has previously been shown that percentage infectivity correlates well with infectivity/ng p24 in this system.^25^ The PI drugs used in this study were obtained from the AIDS Research and Reference Reagent Program, Division of AIDS, NIAID, NIH.

### Ethics

Informed consent was obtained from all subjects and ethics approval for virological testing was obtained from the National Research Ethics Committee of Nigeria (NHREC/01/01/2007).

### Statistical analysis

Differences in PI susceptibility were compared with the Wilcoxon rank-sum test (GraphPad Software Inc., La Jolla, CA, USA), which is robust to data that are not normally distributed.

## Results

Six matched pairs of patients were included. Table 1 contains clinical and laboratory data on cases, who experienced virological failure (duration), and controls, who suppressed viral replication for 48 weeks. Of note, all pairs but one had a CD4 count <200 cells/mm^3^. All but one pair was treated with lopinavir-based ART (atazanavir was used in one pair). Table 2 shows NRTI and NNRTI resistance mutations detected prior to second-line initiation. All patients had lamivudine resistance [M184V/I in reverse transcriptase (RT)] and 7/12 (58.3%) had at least moderate resistance to tenofovir (3 with K65R, 3 with K70E and 1 with three thymidine analogue mutations including M41L, L210W and T215Y). All 12 individuals had high-level NNRTI resistance. Two pairs were infected with subtype G viruses and four pairs with CRF02_AG viruses (Table 2). No major mutations in protease were observed in the patients. We analysed sequences for mutations in Gag in cases and controls associated with PI susceptibility or exposure (Table 3).

**Table 1.** Clinical data for matched patient pairs comprising virological successes and failures

Sample pair	Age (years)		Sex		Baseline^a^ CD4 count (cells/mm^3^)		Second-line PI used		Baseline^a^ viral load (copies of HIV-1 RNA/mL)	
	success	failure	success	failure	success	failure	success	failure	success	failure
1	33	45	female	female	<200	<200	LPV	LPV	503 951	140 991
2	43	36	female	female	<200	<200	LPV	LPV	39 844	20 178
3	27	26	female	female	<200	<200	LPV	LPV	32 284	271 974
4	47	39	female	female	200–499	200–499	LPV	LPV	228 083	24 693
5	35	40	female	female	<200	<200	ATV	ATV	14 487	274 504
6	34	33	female	female	<200	<200	LPV	LPV	39 929	18 056

LPV, lopinavir; ATV, atazanavir.

a Baseline refers to pre-initiation of second-line therapy.

**Table 2.** NRTI and NNRTI mutations observed at first-line failure, prior to initiation of second-line PI-based ART

		NRTI mutations	NNRTI mutations	Baseline^a^ VL (copies of HIV-1 RNA/mL)		HIV-1 subtype		2L backbone	
				success	failure	success	failure	success	failure
Pair 1	success	M41L, L74LI, M184V, L210W, T215F	K101E, E138Q, G190A	503 951	140 991	CRF02_AG	CRF02_AG	TDF/FTC	TDF/FTC
	failure	M184V	K103N						
Pair 2	success	E44D, D67N, T69D, K70R, M184V, T215Y	K101E, K103N	39 844	20 178	CRF02_AG	CRF02_AG	TDF/FTC	TDF/FTC
	failure	M184V	K101E, G190A						
Pair 3	success	K70E, M184V	A98G, Y181C	32 284	271 974	G	G	TDF/FTC	TDF/FTC
	failure	K70E, M184V	Y181C, G190A, H221Y						
Pair 4	success	K70E, Y115F, M184V	K103N	228 083	24 693	CRF02_AG	CRF02_AG	AZT/3TC	AZT/3TC
	failure	K65R, M184V	K101E, V108I, Y181C, G190A						
Pair 5	success	D67N, K70R, M184V, T215F, K219E	Y188C	14 487	274 504	G	G	TDF/FTC	TDF/FTC
	failure	K70R, M184V, K219Q	K103N, Y318F						
Pair 6	success	K65R, M184I	K103N, Y181C	39 929	18 056	CRF02_AG	CRF02_AG	TDF/FTC	TDF/FTC
	failure	K65R, M184I	K103N, Y181C						

AZT, zidovudine; 3TC, lamivudine; FTC, emtricitabine; TDF, tenofovir disoproxil fumarate.

a Baseline refers to pre-initiation of second-line therapy.

**Table 3.** Variation in Gag cleavage and non-cleavage sites and protease

	HIV-1 subtype	Treatment outcome	Gag cleavage sites					Gag non-cleavage sites
			*MA/CA 128–137 VSQNY/ PIVQN*	*CA/p2 359–368 KARVL/ AEAMS*	*p2/NC 373–382 SATIM/ MQRGN*	*NC/p1 428–437 ERQAN/ FLGKI*	*p1/p6 444–453 RPGNF/ LQSRP*	
Pair 1	CRF02_AG	success	*-----/-----*	*-----/-----*	*-+N--/-----*	*-----/-----*	*-----/P----*	*E12K*, *R76K*, *R79F*, *H219Q*, *T242N*, *R409K*, *T487S*
		failure	*-----/-----*	*-----/-----*	*T +N--/-----*	*-----/-----*	*-----/P----*	*R76K*, *R79F*, *T81A*, *G248A*, *V370A*, *R409K*, *E468K*, *T487S*
Pair 2	G	success	*-----/-----*	*-----/-----*	*A-A--/-----*	*-----/----L*	*-----/--N--*	*E12K*, *R76K*, *R79F*, *G123E*, *H219Q*, *G248A*, *V370A*, *R409K*, *T487S*
		failure	*-----/-----*	*-----/-----*	*---+-/---S-*	*-----/-----*	*-----/--N-L*	*E12K*, *G62R*, *R76K*, *H219Q*, *V370A*, *R409K*, *T487S*
Pair 3	G	success	*-----/-----*	*-----/-----*	*--A--/--KS-*	*-----/-----*	*-----/--N-L*	*E12K*, *G62R*, *R76K*, *H219Q*, *T242N*, *G248A*, *V370A*, *R409K*, *T487S*
		failure	*-----/-----*	*-----/-----*	*-+NV-/--K--*	*-----/-----*	*-----/P----*	*E12K*, *R76K*, *R79F*, *H219Q*, *T242N*, *V370A*, *T371del*, *R409K*, *T456S*, *T487S*
Pair 4	CRF02_AG	success	*+----/-----*	*-----/-----*	*T+NV-/--K--*	*-----/-----*	*-----/P----*	*R76K*, *R409K*, *T487S*
		failure	*+----/-----*	*-----/-----*	*-+NV-/-K--*	*-----/-----*	*-----/P----*	*E12K*, *R76K*, *R79F*, *G248A*, *V370A*, *R409K*, *T487S*
Pair 5	G	success	*-----/-----*	*-----/-----*	*A-A--/-----*	*-----/---R-*	*-----/-----*	*E12K*, *R76K*, *R79F*, *G123E*, *V370A*, *R409K*, *T487S*
		failure	*+---F/-----*	*-----/-----*	*QPN--/I----*	*-----/----V*	*-----/P----*	*E12K*, *R76K*, *R79F*, *S165N*, *H219Q*, *V370A*, *R409K*, *T487S*
Pair 6	CRF02_AG	success	*S----/-----*	*---I-/-----*	*ANI--/-----*	*-----/-----*	*-----/P----*	*E12K*, *R76K*, *R79F*, *V370A*, *R409K*, *T487S*
		failure	*+----/-----*	*-----/-----*	*T+NV-/--K--*	*-----/-----*	*-----/P----*	*E12K*, *G248A*, *V370A*, *R409K*, *S451T*, *T487S*

The consensus clonal sequence at each of the Gag cleavage sites is shown for the six pairs using HXB2 numbering. Deletions are represented by +.

**Figure 1.**
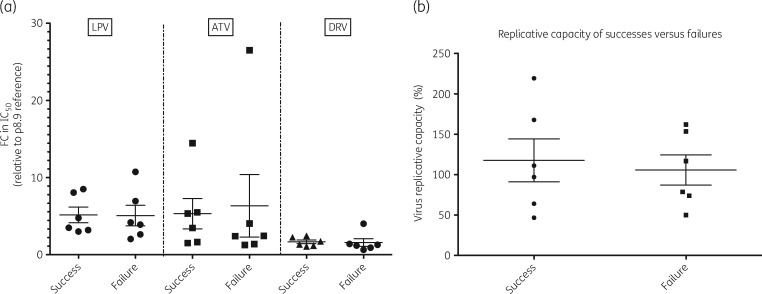
(a) PI susceptibility relative to a subtype B reference strain, expressed as FC in IC_50_, and (b) single-round replication efficiency (relative to a subtype B reference strain) of patient-derived *gag-protease*-containing pseudoviruses derived from patients prior to initiation of second-line boosted PI treatment who either did (Success) or did not (Failure) suppress viral replication after 48 weeks. Each data point is the mean of at least two independent experiments and hairs represent mean and SD. LPV, lopinavir; ATV, atazanavir; DRV, darunavir.

The median (IQR) lopinavir fold change (FC) was 4.04 (2.49–7.89) for virological failures and 4.13 (3.14–8.17) for virological successes (*P *=* *0.94), as described in Figure 1(a). The median (IQR) atazanavir FC was 2.43 (1.35–9.66) for virological failures and 4.39 (1.60–7.73) for successes (*P *=* *0.47). The median (IQR) darunavir FC was 1.234 (0.84–2.05) for virological failures and 1.529 (1.14–2.319) for successes (*P *=* *0.47).

One patient had an FC >10 for lopinavir at baseline and experienced subsequent virological failure on boosted lopinavir as the PI. We also measured the single-round replication efficiency of patient-derived *gag-protease*-containing pseudoviruses derived prior to initiation of second-line boosted PI treatment from patients who either did (success) or did not (failure) suppress viral replication after 48 weeks (Figure 1b). Mean replication efficiency relative to a subtype B reference strain was 117.7% for the successes and 105.8% for failures. There was no statistically significant difference in replication efficiency between the two groups (*P *=* *0.93 by Mann–Whitney *U*-test).

**Figure 2.**
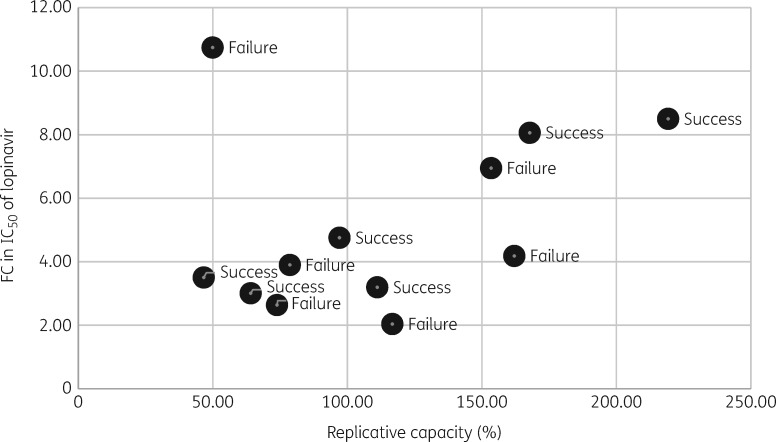
Scatter plot of FC in IC_50_ of lopinavir relative to a subtype B reference strain, versus replicative capacity as measured over a single round of replication (also relative to a subtype B reference strain, which is represented by 100%).

Finally, we analysed the relationship between single-round replication efficiency and FC to lopinavir in all viruses tested. There was a moderate correlation between these parameters (correlation coefficient 0.32, Figure 2). When a single outlier was excluded from analysis (FC 10.7 with replication efficiency 50.0%), the correlation coefficient increased to 0.78.

## Discussion

Given the contribution of the highly polymorphic Gag protein and resulting epistatic interactions to PI susceptibility, we hypothesized that patients would respond differently to these drugs, particularly in the context of extensive NRTI resistance. We previously reported an association between susceptibility to PI and outcome of first-line ritonavir-boosted lopinavir monotherapy in a clinical trial. Here we performed a similar study in patients about to start second-line combination ART, including ritonavir-boosted lopinavir or atazanavir as well as two NRTIs. We found the difference in phenotypic drug susceptibility (assessed by FC relative to a subtype B reference) was not statistically different between the virological failures (cases) and virological successes (controls) for any of the PIs tested: lopinavir, atazanavir or darunavir.

This negative result could be due to the influence of adherence, in that second-line therapy is used in patients for whom first-line therapy has failed, usually as the result of incomplete adherence. Therefore, the patient group was enriched for poor adherers, which could have overcome the effects of small differences in susceptibility.

Interestingly, we previously showed that 2/2 patients with FC >10 prior to PI monotherapy went on to virological failure.^27^ In this study the only patient with FC >10 for lopinavir failed treatment with this drug. Further work needs to be undertaken to explore whether a threshold FC of 10 in our assay is relevant in larger datasets.

We also showed here that replication efficiency over a single round was correlated with lopinavir susceptibility prior to initiation of the bPI. We have previously reported similar findings in replication-competent subtype C viruses that contained patient-derived *gag* and partial *protease* genes.^12^ These data suggest that increased replicative capacity and resistance to PI might involve an overlapping mechanism.

### Limitations

Limitations of our study include the relatively small sample size, the inclusion of more than one subtype and the possibility of viral recombination through our PCR and cloning strategy. In addition, the process of mapping next-generation sequencing reads to a consensus reference sequence to generate a patient consensus can introduce biases against variation, which may affect the identification of novel drug resistance mutations. Finally, our assay system did not incorporate the native gp160 envelope.

Despite introduction of second-generation integrase inhibitors such as dolutegravir as first-line therapy in areas where pre-treatment resistance is >10%,^28^^,^^29^ bPI will still be used as second-line therapy for those who fail dolutegravir-based first-line regimens. Therefore, research into determinants of responses to PI in non-B subtypes is as important as ever.
